# Multichannel high noise level ECG denoising based on adversarial deep learning

**DOI:** 10.1038/s41598-023-50334-7

**Published:** 2024-01-08

**Authors:** Franck Lino Mvuh, Claude Odile Vanessa Ebode Ko’a, Bertrand Bodo

**Affiliations:** 1 Departement of Physics, University of Yaoundé 1, PO.BOX 812, Yaoundé, Cameroon; 2 Yaoundé Gynaecology, Obstetrics and Pediatrics Hospital, PO.BOX 4362, Yaoundé, Cameroon

**Keywords:** Computational biology and bioinformatics, Scientific data

## Abstract

This paper proposes a denoising method based on an adversarial deep learning approach for the post-processing of multi-channel fetal electrocardiogram (ECG) signals. As it’s well known, noise leads to misinterpretations of fetal ECG signals and thus limits the use of fetal electrocardiography for healthcare applications. Therefore, denoising algorithms are essential for the exploitation of non-invasive fetal ECG. The proposed method is based on the combination of three end-to-end trained sub-networks to convert noisy fetal ECG signals into clean signals. The first two sub-networks are linked by skip connections and form a deep convolutional network that downsamples the noisy signals into a latent representation and subsequently upsamples this latent representation to recover clean signals. The third sub-network aims to boost the decoder sub-network to generate realistic clean signals. Experiments carried out on synthetic and real data showed that the proposed method improved by the signal-to-noise (*SNR*) of fetal ECG signals with input *SNR* ranging from $-\,30$ to 0 dB by an average of 20 dB, and improve fetal signal quality by significantly increasing the number of true detected QRS complexes and halving QRS complex detection errors.

## Introduction

Early information on the condition of fetal heart can help to prevent half of all stillbirths^1^. Fetal monitoring is crucial in providing valuable information for timely intervention and avoiding damage to vital fetal organs. Cardiotocography is a conventional obstetrical monitoring technique used to assess fetal distress by processing the pulse and uterine contractions recorded simultaneously from the fetus and the mother respectively^2^. Although cardiography is a standard, non-invasive technique for fetal heart monitoring, the signals recorded do not always reflect abrupt changes in fetal heart rate^3,4^. This can lead to misinterpretation and misdiagnosis, and therefore devalues cardiotocography clinically^5^.

To accurately diagnose fetal cardiac pathologies, the morphology of the electrocardiographic signal is required in the early stages of pregnancy to enrich the information provided by cardiotocography^3,6^ and other sonographic techniques^7–9^. Electrocardiography techniques in which an electrode is placed directly on the fetal scalp can provide this morphological information. The electrocardiogram (ECG) obtained on the scalp is more reliable for monitoring the fetal heart, but it can only be recorded during labor after the amniotic fluid membrane has ruptured, and may pose risks of infection for both fetus and mother^10^. Non-invasive fetal electrocardiography, which involves placing electrodes on the maternal abdomen, offers a promising alternative to standard fetal monitoring methods^11–13^. Combining all the advantages of the aforementioned techniques, it is non-invasive, safe, and comfortable for both fetus and mother. It provides more accurate information and can be used during pregnancy, unlike the scalp ECG recording method. However, the use of non-invasive fetal ECG in clinical practice remains limited due to its low signal-to-noise ratio (SNR)^10,14^. Noises mixed with non-invasive fetal ECG that complicated its extraction include instrumental noise, maternal ECG, fetal and maternal electromyographic signals, and motion artifacts^15,16^. These noises can distort the waveform of the fetal ECG, leading to misinterpretation of fetal heart status^17,18^. It is therefore imperative to develop denoising methods that provide ECG signals with an accurate representation of fetal heart status.

To remove noise from fetal ECG signals, several methods have been proposed in the literature. These methods are generally used as post-processing tools after removal of the maternal ECG from the abdominal recordings, in order to improve the quality of the extracted fetal ECG. They can be grouped into model-based methods and learning-based methods. Model-based methods include most classical signal denoising algorithms such as low-pass filtering^19^, non-local means based methods^20,21^, signal decomposition techniques in frequency and time domains, such as spectral decomposition^22,23^, empirical mode decomposition^24,25^ and discrete wavelet transform^26,27^. Most model-based methods are simple to implement, but suffer from their inability to perform well under a variety of noise conditions.

Recently, deep learning-based methods have achieved incredible success in many fields, such as image restoration^28–31^, image segmentation^32–35^, audio filtering^36–39^, and audio classification^40–43^. These success stories have attracted more and more in-depth research in the field of ECG noise reduction. Methods designed for ECG signal quality restoration and enhancement^44–47^, QRS detection^48–51^, and fetal ECG signal extraction^52–56^ are representative examples. Fotiadou et al.^57^ were the first to propose a fully convolutional deep neural network for the post-processing of non-invasive fetal ECG signals recorded both in single-channel and multi-channel configuration. Their method significantly improves the quality of fetal ECG signals and successfully preserves morphological signal changes. However, in the case of channel signals heavily corrupted by noise, it was not able to reconstruct the relevant morphological features of the fetal ECG, leading to an erroneous diagnosis due to added waves in the reconstructed signals that should not have been there. Singh et al.^58^ proposed a method based on a generative adversarial neural network for denoising adult ECG. Their model showed good generalization ability for multiple noise scenarios but failed in the case of highly corrupted signals.

In this work, to overcome the aforementioned limits, we introduced a deep neural network with skip connections capable of denoising multi-channel fetal ECG signals. The proposed network learns, in a supervised and adversarial way, a sequence of nonlinear transformations that can take the noise-corrupted ECG signals as input and deliver the noise-free ECG signals as output. The trained network eliminates noise in the extracted fetal ECG signals by condensing them into a latent representation, followed by upsampling to recover details.

This paper is organized as follows: In the following section, we explain the proposed approach to fetal ECG denoising and describe the datasets used for training and evaluation of the proposed method. We also describe the other denoising methods used for a comparative study with the proposed method. The “Results and discussion” section is focused on results and discussion, and the paper ends with a conclusion followed by some perspectives.

## Material and methods

### Methodology

#### Proposed approach

Our approach aims to train, in a supervised way, a neural network that denoise highly corrupted fetal ECG signals using a dataset of paired clean and noisy fetal ECG signals. More formally, this problem can be stated as follows:

Given a paired signal training dataset $\left\{ (\hat{x},x)_1, \ldots , (\hat{x},x)_N\right\}$, where $\hat{x}$ represented the noisy version of the clean fetal ECG signal *x* for a given sample, our goal is to learn the right parameter values of a neural network so that the network can map the noisy signal $\hat{x}$ to the clean signal *x*. With these optimized parameters, the neural network can be used to denoise all fetal ECG signals, including those not available in the training database.

**Figure 1:**
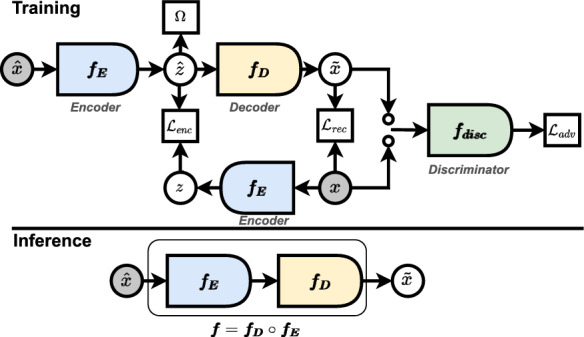
Training and inference schemes of the proposed approach for fetal ECG denoising.

Figure 1 illustrated the overview of the proposed approach at training and inference time.

Three networks involve in training. The first two sub-networks are the encoder and the decoder networks, respectively $f_E$ and $f_D$. They are highlighted in blue and yellow respectively in Fig. 1 and are components of the final network *f* used to denoise the signals at inference time. The last one, highlighted in green, is the discrimnator network ($f_{disc}$).

The encoder network ($f_E$) is composed of eight dilated convolutional layers. Each one performs a sequence of three operations. These operations are a 1D convolution, a leaky rectified linear unit (leakyReLU) activation function, and an instance normalization operations. The encoder downscales the input noisy fetal ECG signal $\hat{x}$, compressing it to a vector of lower dimensions $\hat{z}$, hypothesized to live in space of the best features of *x* and thus referred to as the latent vector of *x*. That means by mapping $\hat{x}$ to $\hat{z}$, $f_E$ only retains useful features of the signal and thus ignores useless features as noise. The decoder network upscales the latent representation $\hat{z}$ to a signal $\tilde{x}$ with the same dimension as *x* for consistent comparison. It consists of nine layers. The first eight perform a sequence of three operations each. These operations are the same as those performed in the encoder layer, except that the dilated convolution is replaced by a transposed convolution operation. The last layer of the decoder is a regular convolution layer performing a convolution operation with no activation function or normalization operation. The discriminator network ($f_{disc}$) is composed of four layers, each ones performing a regular linear operation followed by a rectified linear unit (ReLU) operation. except the last one in which . Its role is to distinct the $\tilde{x}$ signal generated by the decoder from the clean signal *x*. The $f_{disc}$ network is the part responsible for the adversarial learning in the proposed approach.

**Figure 2:**
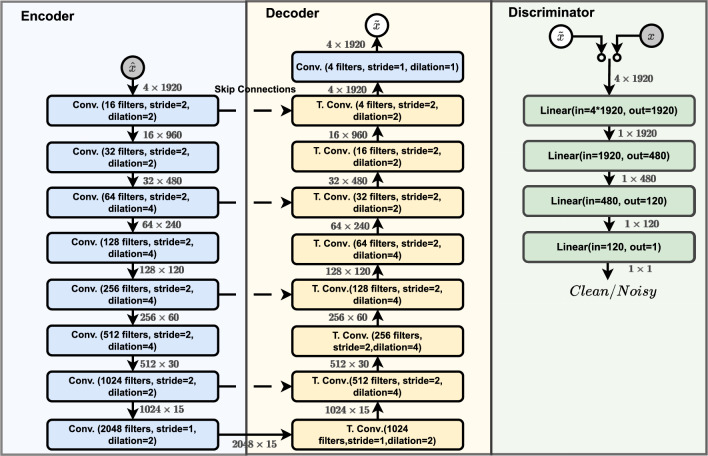
The Encoder, Decoder, and Discriminator architectures of the proposed approach.

Figure 2 depicts the architectures of the encoder, decoder and the discriminator networks. This figure shows that every two convolutional and mirrored transposed convolutional layers are linked with dashed arrows. These dashed arrows are known as “skip” connections and help to deal with the gradient vanish problem occurring in deep architectures^59^ such as the one proposed. The input dimensionality of features at each layer is also presented in Fig. 2, and a more detailed view with the output dimensionality, the number of trainable parameters and all the variables taken into account by the different operations at each layer is shown in Table 1.

During training, the encoder learns to retain the useful features of a noisy input signal $\hat{x}$ by mapping it to a much lower-dimensional vector $\hat{z}=f_E(\hat{x})$. In addition, the encoder is again used to map the clean signals *x* to the corresponding latent lower-dimensional vector $z=f_E(x)$. Since *z* and $\hat{z}$ are of equal dimension, they can be used for consistent comparison, thus enhancing the encoder’s ability to eliminate unnecessary features when encoding noisy signals. This second use of the encoder is one of the unique aspects of the proposed approach. The decoder takes the latent vector $\hat{z}$ as input and learns to generate a signal $\tilde{x}=f_D(\hat{z})$ as close as possible to the clean signal *x*. The generated signal and the clean signal are then fed into the discriminator which, by discriminating between the generated signal and the clean signal, helps the decoder to generate a better signal which finally fools the discriminator. The loss functions for training the respective networks are presented in the next section.

The convolution operation in the encoder and the decoder networks uses a dilation factor to increase the perceptual field of our networks so that the temporal structure of the noisy input ECG signal can be captured at multiple scales. In addition, the encoder network is designed to handle a four-channel ECG signal, enabling the spatial information provided by each channel to be exploited. In this way, spatio-temporal information is exploited to eliminate noise and provide clean signals while preserving the morphology of the individual ECG components.

Based on the above, after training, the encoder and decoder networks can be used to design the final filter *f* defined as $f=f_D \circ f_E$ for denoising unseen noisy fetal ECG.

**Table 1:** Detailed description of the proposed networks architectures.

Network	Layer	Output shape	Kernel size	Parameters
Encoder	Convolution(s = 2, d = 2)	$16\times 960$	8	528
	LeakyReLU(0.2) + IN	$16\times 960$	–	32
	Convolution(s = 2, d = 2)	$32\times 480$	8	4128
	LeakyReLU(0.2) + IN	$32\times 480$	–	64
	Convolution(s = 2, d = 4)	$64\times 240$	8	16448
	LeakyReLU(0.2) + IN	$64\times 240$	–	128
	Convolution(s = 2, d = 4)	$128\times 120$	8	65664
	LeakyReLU(0.2) + IN	$128\times 120$	–	256
	Convolution(s = 2, d = 4)	$256\times 60$	8	262400
	LeakyReLU(0.2) + IN	$256\times 60$	–	512
	Convolution(s = 2, d = 4)	$512\times 30$	8	1049088
	LeakyReLU(0.2) + IN	$512\times 30$	–	1024
	Convolution(s = 2, d = 2)	$1024\times 15$	8	4195328
	LeakyReLU(0.2) + IN	$1024\times 15$	–	2048
	Convolution(s = 1, d = 2)	$2048\times 15$	8	16779264
	LeakyReLU(0.2) + IN	$2048\times 15$	–	4096
Decoder	Transposed Convolution(s = 1, d = 2)	$1024\times 15$	8	16778240
	LeakyReLU(0.2) + IN	$1024\times 15$	–	2048
	Transposed Convolution(s = 2, d = 4)	$512\times 30$	8	8389120
	LeakyReLU(0.2) + IN	$512\times 30$	–	1024
	Transposed Convolution(s = 2, d = 4)	$256\times 60$	8	1048832
	LeakyReLU(0.2) + IN	$256\times 60$	–	512
	Transposed Convolution(s = 2, d = 4)	$128\times 120$	8	524416
	LeakyReLU(0.2) + IN	$128\times 120$	–	256
	Transposed Convolution(s = 2, d = 4)	$64\times 240$	8	65600
	LeakyReLU(0.2) + IN	$64\times 240$	–	128
	Transposed Convolution(s = 2, d = 2)	$32\times 480$	8	32800
	LeakyReLU(0.2) + IN	$32\times 480$	–	64
	Transposed Convolution(s = 2, d = 2)	$16\times 960$	8	4112
	LeakyReLU(0.2) + IN	$16\times 960$	–	32
	Transposed Convolution(s = 2, d = 2)	$4\times 1920$	8	1028
	LeakyReLU(0.2) + IN	$4\times 1920$	–	8
	Convolution(s = 1, d = 1)	$4\times 1920$	8	132
Discriminator	Linear(in = 7680, out = 1920)	$1\times 1920$	–	14747520
	ReLU	$1\times 1920$	–	–
	Linear(in = 1920, out = 480)	$1\times 480$	–	922800
	ReLU	$1\times 480$	–	–
	Linear(in = 480, out = 120)	$1\times 120$	–	57720
	ReLU	$1\times 120$	–	–
	Linear(in = 120, out = 1)	$1\times 1$	–	121
	Sigmoid	$1\times 1$	–	–
s and d stand respectively for stride and dilation parameters, IN stands for Instance Normalization				

#### Objective function and training parameters

To train the networks parameters of the proposed approach, we formulate an objective function by combining three loss functions, each optimizing the individual sub-networks of the proposed approach.

The first is a contractive denoising auto-encoder loss $\mathscr{L}_{ctr}$ introduced in Refs.^60,61^ and used in Ref.^62^ for ECG filtering purposes. It is formed by the addition of two terms, a classical denoising auto-encoder loss $\mathscr{L}_{rec}$ computing the mean squared error (MSE) between the clean fetal ECG and the denoised fetal ECG, and a Frobenius norm $\Omega$ of Jacobian matrix computed from the noisy input fetal ECG. Hence, the contractive loss $\mathscr{L}_{ctr}$ is defined as follows:

1 $$\begin{aligned} \mathscr{L}_{ctr} = \mathscr{L}_{rec} + \Omega \end{aligned}$$

with

2 $$\begin{aligned} \mathscr{L}_{rec} = \left\| x-\tilde{x}\right\| ^2_2 \end{aligned}$$

and,

3 $$\begin{aligned} \Omega = \left\| \frac{\partial f_E(\hat{x})}{\partial \hat{x}} \right\| ^2_F \end{aligned}$$

The next is responsible for adversarial learning introduced in generative adversarial networks (GANs)^63^. The GAN framework commonly comprises two sub-networks, a generator network and a discriminator, with competing objectives^63^. They have achieved considerable success in image processing and are still being explored for time series applications such as time classification and synthesis^64–68^. However, it has been shown that many GAN-based algorithms trained with classical adversarial loss may not converge^63,69^. To avoid this problem and continue benefit from the advantages offered by GANs, several techniques for computing loss functions have been introduced to encourage GAN-based algorithms convergence^70–72^. We use the one called feature matching proposed in Ref.^70^, which uses the internal representation of the discriminator network to update the generator parameters. In our reformulation of the GAN framework, the decoder network plays the role of the generator network, hence the adversarial loss $\mathscr{L}_{adv}$ is defined as the MSE between the feature representation of the clean fetal ECG and the generated fetal ECG, respectively:

4 $$\begin{aligned} \mathscr{L}_{adv} = \left\| f_{disc}(x)-f_{disc}\left( f_D(\hat{z})\right) \right\| ^2_2 \end{aligned}$$

where $f_{disc}$ is a function that outputs an intermediate layer of the discriminator network.

The $\mathscr{L}_{ctr}$ and $\mathscr{L}_{adv}$ introduced above encourage the neural network *f*, especially the decoder part $f_D$ to produce realistic and similar signals to clean fetal ECG. This work can be done only under the condition that the encoder part produces a vector $\hat{z}$ capturing the best representations of *x*. To enforce that, we introduced an additional loss function $\mathscr{L}_{enc}$ defined as the MSE between the latent vector of the clean fetal signal and the latent vector of the noisy fetal signal:

5 $$\begin{aligned} \mathscr{L}_{enc} = \left\| f_E(x)-f_E(\hat{x})\right\| ^2_2 \end{aligned}$$

Overall, the objective function to train the proposed approach is defined as the weighted average of the above loss functions:

6 $$\begin{aligned} \mathscr{L} = w_{enc}\mathscr{L}_{en} + w_{adv}\mathscr{L}_{adv} + w_{rec}\mathscr{L}_{rec} + w_{\omega }\Omega \end{aligned}$$

where $w_{enc}$, $w_{adv}$, $w_{rec}$, and $w_{\omega }$ are the hyper-parameters adjusting the contribution of individual losses to the overall objective function. Since each fetal signal has four channels, the overall objective is computed along each channel and then averaged.

In our experiments, we organized signals in batches of size $B=8$ and used as optimization algorithm the variant of the Adam algorithm proposed in Ref.^73^ with weight decay and learning rate parameters set to $5.10^{-2}$ and $10^{-5}$, respectively. We empirically found that setting the weighting parameters $w_{enc}$, $w_{adv}$, $w_{rec}$, $w_{\omega }$ to 4, $10^{-2}$, 25, $10^{-4}$, respectively, allowed fast convergence. The network was implemented in Pytorch and trained for 20 epochs on a 64-bit Intel Core i7 processor with 8 GB of RAM.

### Data for training and evaluation

Two datasets were used for training purposes and to evaluate the proposed method, as well as the other methods chosen for benchmarking. The first one is a synthetic fetal ECG dataset created by employing the open-source fecgsyn toolkit developed in Ref.^74^. The fecgsyn toolbox is used to create a dataset suitable for network training by dividing each sample in the dataset into an abdominal noisy signal and a fetal ECG clean signal of thirty-two channels each. Using the fecgsyn toolbox, we simulate different physiological event cases with different noise levels ranging from $-\,12$ to 12 dB and from $-\,30$ to 0 dB respectively, for training and evaluation purposes. The physiological event cases considered are described in Table 2, they are similar to events simulated in the Fetal ECG Synthetic Database^74,75^ except that we excluded the case of twin pregnancy as the proposed model was not developed to handle this case. Each simulation was run, for statistical purposes, five times independently using a five-minute signal at a sampling rate of 250 Hz.

The second one is an open-access dataset called the Abdominal and Direct Fetal Electrocardiogram Database^76^. It consists of real multi-lead abdominal fetal ECG signals obtained from five women in labor, between 38 and 41 weeks of gestation. Each signal has been recorded with 16-bit resolution at a sampling rate of 1000 Hz for five minutes and was processed by digital filters to eliminate baseline and power-line inference^76^. For each record, four-channel abdominal mixtures and the corresponding direct fetal ECG signals are given.

**Table 2:** Description of physiological events of the synthetic signals of synthetic dataset.

Case	Description
Baseline	Abdominal mixture (no noise)
Case 0	Baseline signals with noise
Case 1	Baseline signals with fetal movement
Case 2	Baseline signals with heart rate acceleration
Case 3	Baseline signals with uterine contraction
Case 4	Baseline signals with ectopic beats

### Data preprocessing

Before applying the proposed algorithm to data, certain preprocessing steps are necessary. Firstly, using the method based on the Extended Kalman Filtering^77^, the cancellation of the maternal ECG was performed for the abdominal part of all recordings in the synthetic and real databases.

Then, for each thirty-two channel signal in the synthetic dataset, eight channels were selected to form two four-channel signals. The channels were selected according to recommendations given in Ref.^74^. All channels were taken into account in the real database since there are only four. Finally, all the fetal ECG signals were resampled to 500 Hz to have a common frequency and they were divided into sequences of $4 \times 1920$ samples. The signals were also standardized along each channel to have a mean of zero and a unit standard deviation.

### Performance evaluation

#### Performance metrics

In this study, two sets of performance measures were used to evaluate the proposed approach. The first set measures the divergence between the signal output by the proposed method and the ground truth signal. These measures were only applied to the synthetic dataset, since for each noisy fetal ECG in the synthetic dataset, its clean version is available. These metrics include the signal-to-noise ratio improvement (*SNR*$_{imp}$), the root mean-square error (*RMSE*) and the percent-root distortion (*PRD*).

*SNR*$_{imp}$ measures the difference of *SNR* between a denoised signal and the corresponding noisy input signal. A higher value of *SNR*$_{imp}$ indicates better denoising performance. It can be expressed for a channel, *c*, of a signal as follows:

7 $$\begin{aligned} \textit{SNR}_{imp} = \textit{SNR}_{out} - \textit{SNR}_{in} \end{aligned}$$

where *SNR*$_{in}$ and *SNR*$_{out}$ are defined as:

8 $$\begin{aligned} \textit{SNR}_{in}= & {} 10\times \log _{10}{\left( \frac{\sum _{t=1}^T x_{c,t}^2}{\sum _{t=1}^T (\hat{x}_{c,t}-x_{c,t})^2}\right) } \end{aligned}$$

9 $$\begin{aligned} \textit{SNR}_{out}= & {} 10\times \log _{10}{\left( \frac{\sum _{t=1}^T x_{c,t}^2}{\sum _{t=1}^T (\tilde{x}_{c,t}-x_{c,t})^2}\right) } \end{aligned}$$

*RMSE* measures the variance between the denoised signal and the corresponding clean signal. A lower value of *RMSE* corresponds to a smaller difference and is desired as it indicates better performance. For a channel, *c*, of a signal, *RMSE* is defined as:

10 $$\begin{aligned} \textit{RMSE} = \sqrt{\frac{1}{T}\sum _{t=1}^T (x_{c,t}-\tilde{x}_{c,t})^2} \end{aligned}$$

*PRD* can be used as an indicator of the recovery quality of the compressed signal. A lower value of *PRD* indicates a better quality of the denoised signal. For a channel, *c*, of a signal, it is defined as:

11 $$\begin{aligned} \textit{PRD} = 100\times \sqrt{\frac{\sum _{t=1}^T (x_{c,t}-\tilde{x}_{c,t})^2}{\sum _{t=1}^T x_{c,t}^2}} \end{aligned}$$

These metrics are computed for each channel and then averaged.

In a dataset containing the real fetal ECG, the above metrics are useless since there are no clean fetal ECG signals that can be used as ground truth signals. However, the dataset provides the abdominal recording with the simultaneously recorded scalp ECG annotated by the experts^76^. Although recorded directly from the fetal head, scalp measurements contain a considerable amount of noise. Moreover, they are recorded on a different lead than the abdominal leads; thus, even in the case of perfect denoising, the denoised signals and the scalp signals can not be matched. Nevertheless, the manifestations of major cardiac electrical events such as ventricular depolarization should coincide and be aligned in the ECG signals from the abdomen and scalp. It is therefore possible to base the evaluation of the proposed method on the correctness of detecting the QRS complexes. The calculation of the correctly detected QRS complexes is performed with a tolerance of $\pm \,50$ milliseconds of the scalp R-peak location^78^. Based on correct and incorrect detection, the following metrics can be defined:

12 $$\begin{aligned} \textit{PPV}= & {} \frac{\textit{TP}}{\textit{TP}+\textit{FP}} \end{aligned}$$

13 $$\begin{aligned} \textit{SE}= & {} \frac{\textit{TP}}{\textit{TP}+\textit{FN}} \end{aligned}$$

14 $$\begin{aligned} \textit{F}_1= & {} \frac{2 \textit{PPV}\cdot \textit{SE}}{\textit{PPV} + \textit{SE}} \end{aligned}$$

where *TP* is True Positive, i.e. the correctly detected QRS complexes, *FN* is False Negative, i.e. the undetected QRS complexes; and *FP* is False Positive, i.e. the incorrectly detected QRS complexes. *PPV* measures the accuracy of the detection algorithm by evaluating the ratio of detections of true-positive QRS complexes to the total number of QRS complexes detected; *SE* measures the ability of the detection algorithm to detect true-positive QRS complexes relative to the total number of QRS complexes annotated, and $\textit{F}_1$ is the harmonic mean of sensitivity and accuracy, providing a balanced measure of the performance of the detection algorithm. These metrics are widely used to evaluate QRS complex detection and higher values of them are indicators of better performance denoising^48,51^.

#### Comparative methods

A benchmarking study is carried out to evaluate the proposed approach against three other denoising methods. The first comparative method is wavelet-based. The wavelet transform is widely studied and used for ECG signal analysis because, by expanding the signal in terms of a localized wavelet function in both time and frequency, it provides good time resolution at high frequency and good frequency resolution at low frequency^79^. Wavelet-based denoising methods consist of three main steps consisting of decomposing the signal into coefficients, comparing these coefficients with a certain threshold, and reconstructing the signal with the thresholded coefficients. We selected the sixth-order symlet wavelet as the mother wavelet because it works well on ECG noise^80^ and the sureshrink method was used for thresholding^81,82^.

The next comparative method is a supervised learning-based method. It is a denoising convolutional autoencoder (FCN-DAE) proposed in Ref.^83^ for noise reduction in ECG signals. The architecture of FCN-DAE has three main parts; the encoder part and decoder part using convolution and deconvolution operations together with batch normalization and exponential linear unit (ELU) as activation function, respectively^84^. The last part is the output layer performing only a deconvolution operation. A more exhaustive description can be found in Ref.^83^. It is important to note that FCN-DAE was originally proposed for single-channel ECG filtering^83^, so we slightly modified it to adapt it to four-channel signals configuration.

The last method is the supervised learning-based method designed in^57^, especially for denoising multichannel fetal ECG. It is a deep convolutional neural network (DCNN) consisting of an encoder of eight convolutional layers symmetrically connected with eight transposed convolutional layers of a decoder. The encoder’s layers perform convolution operations such that the signal is downsampled by two after each layer, while the decoder’s layers perform transposed convolutions that upsample the signal by two after each layer. The leaky rectified linear units with a slope of 0.2 are used as a non-linearity operation at each layer. A more exhaustive description of this method can be found in Ref.^57^.

For a fair comparative study, it is important to note that the comparative denoising methods and the proposed method do not have the same complexity. The calibration of wavelet-based methods relies solely on the choice of wavelet family and order, and the choice of threshold, and is done through a trial-and-error process, usually in a short time. Methods based on deep learning require more parameters, more computational resources and more time to train these parameters. The proposed model uses 49,229,492 parameters to estimate the denoised signal, while the DCNN model uses 93,649,796 parameters, just under twice as many, for the same task. It should be noted, however, that training of the proposed model is more complex due to the dual use of the encoder and the presence of the discriminator (Fig. 2). The FCN-DAE model is the simplest of the deep learning models used in this study. It has 86,211 parameters and uses no residual connection like the other two.

## Results and discussion

### Evaluation on synthetic dataset

**Figure 3:**
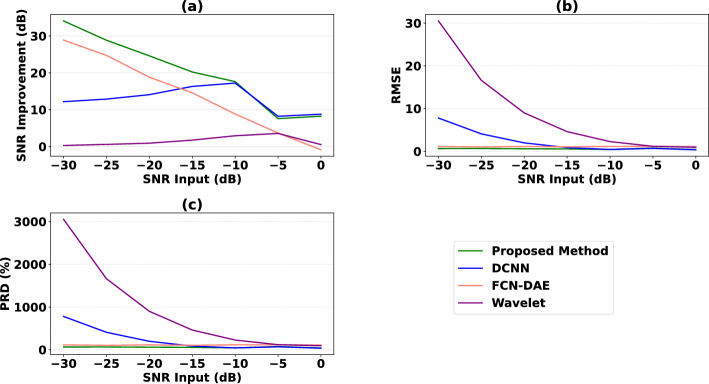
Performance of the proposed network in comparison with the existing methods in terms of (**a**) the improvement in signal-to-noise ratio (*SNR*$_{imp}$), (**b**) the root mean-square-error (*RMSE*), and (**c**) the percent root distortion (*PRD*). Each metric was computed along each channel and then averaged.

The proposed method and the existing methods were evaluated on the synthetic dataset, and the results are presented in Fig.  3. Figure 3 illustrates the improvement in *SNR*, the *RMSE* and the *PRD* for input *SNR* ranging from $-\,30$ to 0 dB. It can be observed that the proposed method outperforms the existing methods by providing the highest values of $\textit{SNR}_{imp}$ and the lowest values of *RMSE* and *PRD* throughout the whole range of input $\textit{SNR}$. More explicitly, for input *SNR* between $-\,10$ and 0 dB the proposed method and the DCNN method^57^ have comparable performances, while the performances of the FCN-DAE method^83^ and the wavelet denoising method gradually become similar. As the input *SNR* decreases, the DCNN and wavelet denoising methods perform less well, rendering them useless in cases of very low-quality signals, while the FCN-DAE method still performs well but not as well as the proposed method.

**Figure 4:**
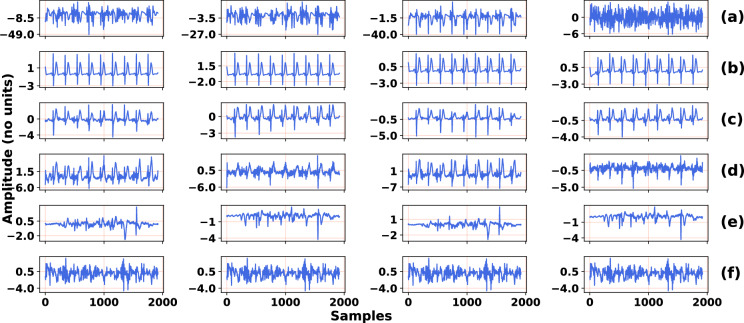
Qualitative comparison of denoising a synthetic signal from the test dataset by the proposed and existing denoising methods. (**a**) the noisy input fetal ECG, (**b**) the ground truth fetal ECG. The signal produced by (**c**) the proposed method, (**d**) the DCNN method, (**e**) the FCN-DAE method, (**f**) the wavelet method. The *SNR* values before and after applying denoising methods are given in Table 3.

Figure 3 shows that the wavelet denoising method gives the worst results. This is not surprising because, in the presence of heavily noisy fetal ECG signals, the wavelet denoising method is not able to preserve individual variations between ECG complexes and is inclined to distort the signal amplitude, whereas the proposed and existing learning-based methods, DCNN and FCN-DAE, achieve better results. Figure 4 illustrates this phenomenon for a typical signal from our synthetic test dataset. The values of performance metrics before and after denoising are provided in Table 3. It can be seen that the signal output by the proposed method is clearer and closer to the ground truth signal than the signal output by other denoising methods. For this particular example, the *SNR* result of the proposed method is around 5 dB higher than the *SNR* result of the second best method, FCN-DAE.

These results clearly show that the proposed method significantly improves the quality of the noisy fetal ECG signals. It preserves morphology, amplitude, and variations among individual fetal ECG complexes, even when most signal channels are severely corrupted. This result is not surprising, and is explained by the loss function (Rq. 5), which explicitly forces the network to capture the best representations of the fetal ECG signal by examining both the noisy fetal ECG and the clean fetal ECG. Moreover, the multi-lead configuration of the input signal enables the network to capture spatio-temporal information in the fetal ECG. These information are beneficial in cases of severely corrupted signal channels since the network has sufficient features to recover each channel.

**Table 3:** Quantitative comparison in terms of *SNR* of the signal depicted in Fig. 4, before ($\textit{SNR}_{in}$) and after ($\textit{SNR}_{out}$) denoising.

Channel	$\textit{SNR}_{in}$	$\textit{SNR}_{out}$ (dB)			
	(dB)	Wavelet	FCN-DAE	DCNN	Proposed method
1	$-$ 20	$-$ 4.87	$-$ 0.54	$-$ 4.36	4.38
2	$-$ 16	$-$ 4.92	$-$ 1.33	$-$ 0.42	3.75
3	$-$ 20	$-$ 4.95	$-$ 0.63	$-$ 3.86	4.86
4	$-$ 8	$-$ 5.11	$-$ 1.24	$-$ 0.56	3.20
Average	$-$ 16	$-$ 4.96	$-$ 0.94	$-$ 2.30	4.05

### Evaluation of real dataset

**Figure 5:**
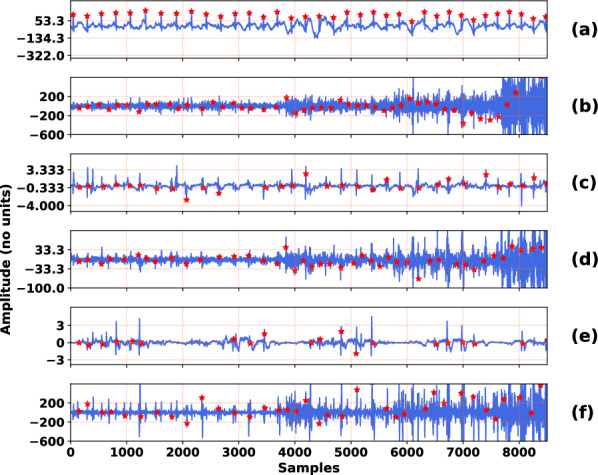
Qualitative comparison of the peak detection over the proposed and existing denoising output samples. In (**a**) the fetal scalp ECG, (**b**) the noisy input fetal ECG. The output signal by (**c**) the proposed method, (**d**) the DCNN method, (**e**) the FCN-DAE method, (**f**) the wavelet method. Only the first channels of the output signal are displayed for better visualization.

As previously stated, the denoising performance of the proposed and existing methods cannot be directly measured in the case of real fetal ECG signals, as there are no clean reference signals. Instead, we examine the performance of QRS complexes detection facilitation. To quantify the above performance, we applied the denoising methods and then the Hamilton peak detector to the signal extracted from the r10 recording of the real fetal ECG dataset. Only the r10 recording was selected from the five available because, after applying the extraction method, others were too noisy to allow denoising and peak detection.

**Figure 6:**
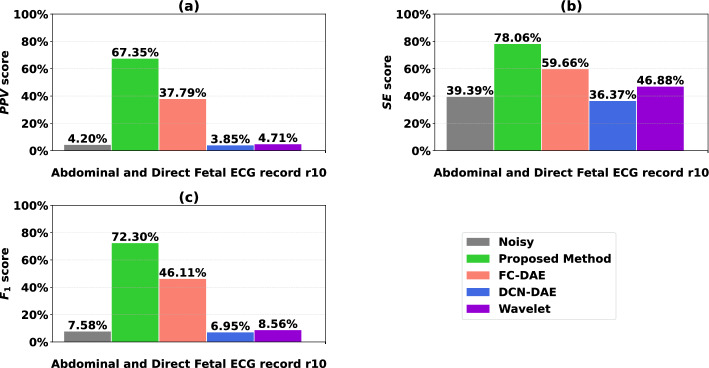
Performance of the proposed method and the existing methods in case of real fetal ECG signals. The considered metrics are (**a**) the positif predicted value (*PPV*), (**b**) the sensitivity (*SE*), and (**c**) the *F*$_1$ score. Each metric was computed along each channel and then averaged.

Figure 5 illustrates a comparison of QRS complexes detection on a piece of extracted noisy fetal ECG and on the denoised signals after the application of the proposed and existing methods. A corresponding piece of scalp ECG is also provided. The red dots in the plots indicate the positions of the QRS complexes detected by the Hamilton peak detector, with the exception of those in the scalp ECG signal, which are provided by the database. For reasons of simplicity and visualization, only the first channels of the denoised signals are shown.

Using the QRS complexes detected on the denoised signals by the Hamilton peak detector and those of the scalp ECG provided by the database, we use the performance metrics described by the equations Eqs. (12), (13), (14) to quantitatively evaluate the methods. These metrics are calculated for each channel and for all samples in record r10 in the real dataset. Figure 6 shows the average values of these metrics, while Table 4 depicts in more detail the values of the performance metrics for each channel.

**Table 4:** QRS complexes detection performance based on Hamilton Peak Detector.

	Channel	***TP***	***FN***	***FP***	***PPV(%)***	***SE(%)***	***F***$_1$(%)
Original fetal ECG	1	26	611	39	40.00	4.08	7.41
	2	24	613	41	36.92	3.77	6.84
	3	27	610	53	33.75	4.24	7.53
	4	30	607	34	46.88	4.71	8.56
Wavelet	1	30	607	34	46.88	4.71	8.56
	2	30	607	34	46.88	4.71	8.56
	3	30	607	34	46.88	4.71	8.56
	4	30	607	34	46.88	4.71	8.56
FCN-DAE	1	244	393	173	58.51	38.30	46.30
	2	258	379	180	58.90	40.50	48.00
	3	203	434	122	62.46	31.87	42.20
	4	258	379	181	58.77	40.50	47.96
DCNN	1	26	611	45	36.62	4.08	7.34
	2	23	614	40	36.51	3.61	6.57
	3	13	624	60	17.81	2.04	3.66
	4	36	601	30	54.55	5.65	10.24
Proposed method	1	433	204	123	77.88	67.97	72.59
	2	428	209	119	78.24	67.19	72.30
	3	445	192	118	79.04	69.86	74.17
	4	410	227	122	77.07	64.36	70.15

These results indicate that the number of true detected QRS complexes (*TP*) and the QRS complexes detection errors (*FP* and *FN*) are respectively increased and reduced over the signal output by the proposed method and the existing methods, with the exception of the DCNN method. The proposed method performs significantly better than the others, achieving the best scores in all performance measures for QRS complex detection with significant difference scores (around $29\%$, $18\%$, $26\%$ difference in *PPV*, *SE* and *F*$_1$ respectively) compared to the second best method, the FCN-DAE. The proposed denoising method considerably increases the number of true detected QRS complexes (*TP*) (over 400 complexes on average) and reduces, on average, the detection errors (*FP* and *FN*) by around $50\%$.

## Conclusion

We have presented a method based on adversarial learning for denoising fetal ECG signals. The proposed denoiser network simultaneously takes as input four fetal ECG leads, encodes them into an ECG latent signal representation, and then decodes this latent representation to recover and output clean and reliable fetal ECG leads. Qualitative and quantitative evaluations on synthetic and real data have shown that the proposed method is superior to the existing ECG denoising methods, such as wavelet-based and learning-based methods, for severely corrupted ECG signals with an SNR value higher than $-\,30$ dB, thus enabling a substantial improvement in the quality of noisy multi-channel fetal ECG signals. All these performances make the proposed method suitable for clinical applications. A future outcome will be the direct denoising of raw abdominal signals, without suppression of the maternal ECG.

## Data Availability

The datasets used and/or analysed during the current study are available from the corresponding author on reasonable request.

## References

[1] Lawn JE, Manandhar A, Haws RA, Darmstadt GL (2007). Reducing one million child deaths from birth asphyxia: A survey of health systems gaps and priorities. Health Res. Policy Syst..

[2] Freeman, R. K., Garite, T. J., Nageotte, M. P. & Miller, L. A. *Fetal Heart Rate Monitoring* 4th edn. (Lippincott Williams and Wilkins, 2012).

[3] Amer-Wåhlin I (2001). Cardiotocography only versus cardiotocography plus ST analysis of fetal electrocardiogram for intrapartum fetal monitoring: A Swedish randomised controlled trial. The Lancet.

[4] Alfirevic Z, Gyte GM, Cuthbert A, Devane D (2017). Continuous cardiotocography (CTG) as a form of electronic fetal monitoring (EFM) for fetal assessment during labour. Cochrane Database Syst. Rev..

[5] Pehrson C, Sorensen J, Amer-Wåhlin I (2011). Evaluation and impact of cardiotocography training programmes: A systematic review. BJOG Int. J. Obstet. Gynaecol..

[6] Vullings R, Van Laar JO (2020). Non-invasive fetal electrocardiography for intrapartum cardiotocography. Front. Pediatr..

[7] Hamelmann P (2019). Doppler ultrasound technology for fetal heart rate monitoring: A review. IEEE Trans. Ultrason. Ferroelectr. Freq. Control.

[8] Fyfe DA, Meyer KB, Case CL (1993). Sonographic assessment of fetal cardiac arrhythmias. Semin. Ultrasound CT MRI.

[9] Adithya PC, Sankar R, Moreno WA, Hart S (2017). Trends in fetal monitoring through phonocardiography: Challenges and future directions. Biomed. Signal Process. Control.

[10] Jagannath D, Selvakumar AI (2014). Issues and research on foetal electrocardiogram signal elicitation. Biomed. Signal Process. Control.

[11] Martin CB (1998). Electronic fetal monitoring: A brief summary of its development, problems and prospects. Eur. J. Obstet. Gynecol. Reprod. Biol..

[12] Smith V (2018). A systematic review of cardiac time intervals utilising non-invasive fetal electrocardiogram in normal fetuses. BMC Pregnancy Childbirth.

[13] Kahankova R (2019). A review of signal processing techniques for non-invasive fetal electrocardiography. IEEE Rev. Biomed. Eng..

[14] Chung CT (2022). Clinical significance, challenges and limitations in using artificial intelligence for electrocardiography-based diagnosis. Int. J. Arrhythm..

[15] Abel JDK, Dhanalakshmi S, Kumar R (2023). A comprehensive survey on signal processing and machine learning techniques for non-invasive fetal ECG extraction. Multimed. Tools Appl..

[16] Zhou Z, Huang K, Qiu Y, Shen H, Ming Z (2021). Morphology extraction of fetal electrocardiogram by slow-fast LSTM network. Biomed. Signal Process. Control.

[17] Pinto P, Costa-Santos C, Gonçalves H, Ayres-De-Campos D, Bernardes J (2015). Improvements in fetal heart rate analysis by the removal of maternal-fetal heart rate ambiguities. BMC Pregnancy Childbirth.

[18] Pavel, M. S. R., Islam, M. R. & Siddiqee, A. M. Fetal arrhythmia detection using fetal ECG signal. In *2019 IEEE International Conference on Telecommunications and Photonics (ICTP)* (IEEE, 2019).

[19] Hermawan, I. *et al.* Denoising noisy ECG signal based on adaptive Fourier decomposition. In *2018 3rd International Seminar on Sensors, Instrumentation, Measurement and Metrology (ISSIMM)* 11–14. 10.1109/ISSIMM.2018.8727739 (IEEE, 2018).

[20] Singh, P. & Pradhan, G. Exploring the non-local similarity present in variational mode functions for effective ECG denoising. In *2018 IEEE International Conference on Acoustics, Speech and Signal Processing (ICASSP)* 861–865. 10.1109/ICASSP.2018.8461768 (IEEE, 2018).

[21] Tracey BH, Miller EL (2012). Nonlocal means denoising of ECG signals. IEEE Trans. Biomed. Eng..

[22] Bonizzi, P., Karel, J., Zeemering, S. & Peeters, R. Sleep apnea detection directly from unprocessed ECG through singular spectrum decomposition. In *2015 Computing in Cardiology Conference (CinC)* 309–312. 10.1109/CIC.2015.7408648 (IEEE, 2015).

[23] Barbosa PRB, Barbosa-Filho J, de Sá CAM, Barbosa EC, Nadal J (2003). Reduction of electromyographic noise in the signal-averaged electrocardiogram by spectral decomposition. IEEE Trans. Biomed. Eng..

[24] Boudraa A-O, Cexus J-C (2007). EMD-based signal filtering. IEEE Trans. Instrum. Meas..

[25] Singh, P., Shahnawazuddin, S. & Pradhan, G. Significance of modified empirical mode decomposition for ECG denoising. In *2017 39th Annual International Conference of the IEEE Engineering in Medicine and Biology Society (EMBC)* 2956–2959. 10.1109/EMBC.2017.8037477(IEEE, 2017). 10.1109/EMBC.2017.8037477 29060518

[26] Awal MA, Mostafa SS, Ahmad M, Rashid MA (2014). An adaptive level dependent wavelet thresholding for ECG denoising. Biocybern. Biomed. Eng..

[27] Shemi, P. M. & Shareena, E. M. Analysis of ECG signal denoising using discrete wavelet transform. In *2016 IEEE International Conference on Engineering and Technology (ICETECH)*. 10.1109/icetech.2016.7569341 (IEEE, 2016).

[28] Haque IRI, Neubert J (2020). Deep learning approaches to biomedical image segmentation. Inform. Med. Unlocked.

[29] Wang S, Yang DM, Rong R, Zhan X, Xiao G (2019). Pathology image analysis using segmentation deep learning algorithms. Am. J. Pathol..

[30] Su J, Xu B, Yin H (2022). A survey of deep learning approaches to image restoration. Neurocomputing.

[31] Zhang K, Zuo W, Chen Y, Meng D, Zhang L (2017). Beyond a Gaussian denoiser: Residual learning of deep CNN for image denoising. Trans. Image Process..

[32] Ronneberger, O., Fischer, P. & Brox, T. U-net: Convolutional networks for biomedical image segmentation. In *Medical Image Computing and Computer-Assisted Intervention–MICCAI 2015* (eds Navab, N. *et al.*) 234–241 (Springer International Publishing, 2015).

[33] Wang G, Ye JC, Man BD (2020). Deep learning for tomographic image reconstruction. Nat. Mach. Intell..

[34] Liu X, Song L, Liu S, Zhang Y (2021). A review of deep-learning-based medical image segmentation methods. Sustainability.

[35] Minaee S (2022). Image segmentation using deep learning: A survey. IEEE Trans. Pattern Anal. Mach. Intell..

[36] Purwins H (2019). Deep learning for audio signal processing. IEEE J. Sel. Top. Signal Process..

[37] Kang Z, Huang Z, Lu C (2022). Speech enhancement using u-net with compressed sensing. Appl. Sci..

[38] Grais, E. M. & Plumbley, M. D. Single channel audio source separation using convolutional denoising autoencoders. In *2017 IEEE Global Conference on Signal and Information Processing (GlobalSIP)* 1265–1269. 10.1109/GlobalSIP.2017.8309164(2017).

[39] Azarang A, Kehtarnavaz N (2020). A review of multi-objective deep learning speech denoising methods. Speech Commun..

[40] Wei S, Zou S, Liao F, Lang W (2020). A comparison on data augmentation methods based on deep learning for audio classification. J. Phys: Conf. Ser..

[41] Alouani, Z., Hmamouche, Y., Khamlichi, B. E. & Seghrouchni, A. E. F. A spatio-temporal deep learning approach for underwater acoustic signals classification. In *2022 18th IEEE International Conference on Advanced Video and Signal Based Surveillance (AVSS)*. 10.1109/avss56176.2022.9959247 (IEEE, 2022).

[42] Noda K, Yamaguchi Y, Nakadai K, Okuno HG, Ogata T (2015). Audio-visual speech recognition using deep learning. Appl. Intell..

[43] Hinton G (2012). Deep neural networks for acoustic modeling in speech recognition: The shared views of four research groups. IEEE Signal Process. Mag..

[44] Hong S, Zhou Y, Shang J, Xiao C, Sun J (2020). Opportunities and challenges of deep learning methods for electrocardiogram data: A systematic review. Comput. Biol. Med..

[45] Arsene, C. T., Hankins, R. & Yin, H. Deep learning models for denoising ECG signals. In *2019 27th European Signal Processing Conference (EUSIPCO)*. 10.23919/eusipco.2019.8902833(IEEE, 2019).

[46] Locher, T., Revach, G., Shlezinger, N., van Sloun, R. J. G. & Vullings, R. Hierarchical filtering with online learned priors for ECG denoising. In *ICASSP 2023—2023 IEEE International Conference on Acoustics, Speech and Signal Processing (ICASSP)*. 10.1109/icassp49357.2023.10095943 (IEEE, 2023).

[47] Kiranyaz S (2022). Blind ECG restoration by operational cycle-GANs. IEEE Trans. Biomed. Eng..

[48] Cai W, Hu D (2020). QRS complex detection using novel deep learning neural networks. IEEE Access.

[49] Belkadi MA, Daamouche A, Melgani F (2021). A deep neural network approach to QRS detection using autoencoders. Expert Syst. Appl..

[50] Teplitzky BA, McRoberts M, Ghanbari H (2020). Deep learning for comprehensive ECG annotation. Heart Rhythm.

[51] Zhong W, Liao L, Guo X, Wang G (2018). A deep learning approach for fetal QRS complex detection. Physiol. Meas..

[52] Mohebbian MR (2022). Fetal ECG extraction from maternal ECG using attention-based cycleGAN. IEEE J. Biomed. Health Inform..

[53] Lee KJ, Lee B (2022). End-to-end deep learning architecture for separating maternal and fetal ECGs using W-Net. IEEE Access.

[54] Zhong W, Liao L, Guo X, Wang G (2019). Fetal electrocardiography extraction with residual convolutional encoder-decoder networks. Australas. Phys. Eng. Sci. Med..

[55] Ghonchi H, Abolghasemi V (2022). A dual attention-based autoencoder model for fetal ECG extraction from abdominal signals. IEEE Sens. J..

[56] Zhong W, Zhao W (2021). Fetal ECG extraction using short time Fourier transform and generative adversarial networks. Physiol. Meas..

[57] Fotiadou E, Vullings R (2020). Multi-channel fetal ECG denoising with deep convolutional neural networks. Front. Pediatr..

[58] Singh P, Pradhan G (2021). A new ECG denoising framework using generative adversarial network. IEEE/ACM Trans. Comput. Biol. Bioinform..

[59] Mao, X., Shen, C. & Yang, Y.-B. Image restoration using very deep convolutional encoder-decoder networks with symmetric skip connections. In *NIPS* Vol. 29 (eds Lee, D. *et al.*) (Curran Associates Inc., 2016).

[60] Rifai, S., Vincent, P., Muller, X., Glorot, X. & Bengio, Y. Contractive auto-encoders: Explicit invariance during feature extraction. In *Proceedings of the 28th International Conference on International Conference on Machine Learning, ICML’11* 833–840 (Omnipress, Madison, WI, USA, 2011).

[61] Qiang Chen, F. *et al.* Contractive de-noising auto-encoder. In *Intelligent Computing Theory* 776–781. 10.1007/978-3-319-09333-8_84 (Springer International Publishing, 2014).

[62] Xiong P (2016). A stacked contractive denoising auto-encoder for ECG signal denoising. Physiol. Meas..

[63] Goodfellow I (2020). Generative adversarial networks. Commun. ACM.

[64] Niu Z, Yu K, Wu X (2020). LSTM-based VAE-GAN for time-series anomaly detection. Sensors.

[65] Brophy E, Wang Z, She Q, Ward T (2023). Generative adversarial networks in time series: A systematic literature review. ACM Comput. Surv..

[66] Kim HY, Yoon JW, Cheon SJ, Kang WH, Kim NS (2021). A multi-resolution approach to GAN-based speech enhancement. Appl. Sci..

[67] Joseph S, Rajan R (2023). Cycle GAN-based audio source separation using time-frequency masking. Circuits Syst. Signal Process..

[68] Festag S, Spreckelsen C (2023). Medical multivariate time series imputation and forecasting based on a recurrent conditional Wasserstein GAN and attention. J. Biomed. Inform..

[69] Goodfellow, I. J. *On Distinguishability Criteria for Estimating Generative Models*. arXiv preprint arXiv:1412.6515 (2014).

[70] Salimans, T. *et al.**Improved Techniques for Training GANs* Vol. 29 (Curran Associates Inc., 2016).

[71] Arjovsky, M., Chintala, S. & Bottou, L. Wasserstein generative adversarial networks. In *In International Conference on Machine Learning* vol. 70, 214–223 (PMLR, 2017).

[72] Li W (2022). *Hausdorff* GAN: Improving GAN generation quality with *Hausdorff* metric. IEEE Trans. Cybern..

[73] Loshchilov, I. & Hutter, F. *Decoupled Weight Decay Regularization*. arXiv preprint arXiv:1711.05101 (2017).

[74] Andreotti F, Behar J, Zaunseder S, Oster J, Clifford GD (2016). An open-source framework for stress-testing non-invasive foetal ECG extraction algorithms. Physiol. Meas..

[75] Goldberger AL (2000). Physiobank, physiotoolkit, and physionet. Circulation.

[76] Jezewski J, Matonia A, Kupka T, Roj D, Czabanski R (2012). Determination of fetal heart rate from abdominal signals: Evaluation of beat-to-beat accuracy in relation to the direct fetal electrocardiogram. Biomed. Tech./Biomed. Eng..

[77] Andreotti F (2014). Robust fetal ECG extraction and detection from abdominal leads. Physiol. Meas..

[78] Warmerdam GJ, Vullings R, Schmitt L, Van Laar JO, Bergmans JW (2018). Hierarchical probabilistic framework for fetal R-peak detection, using ECG waveform and heart rate information. IEEE Trans. Signal Process..

[79] Chatterjee S, Thakur RS, Yadav RN, Gupta L, Raghuvanshi DK (2020). Review of noise removal techniques in ECG signals. IET Signal Process..

[80] Li D, Zhang H, Zhang M (2017). Wavelet de-noising and genetic algorithm-based least squares twin SVM for classification of arrhythmias. Circuits Syst. Signal Process..

[81] KumarRai R, Asnani J, Sontakke TR (2012). Review of shrinkage techniques for image denoising. Int. J. Comput. Appl..

[82] Donoho DL, Johnstone IM (1995). Adapting to unknown smoothness via wavelet shrinkage. J. Am. Stat. Assoc..

[83] Chiang H-T (2019). Noise reduction in ECG signals using fully convolutional denoising autoencoders. IEEE Access.

[84] Clevert, D.-A., Unterthiner, T. & Hochreiter, S. *Fast and Accurate Deep Network Learning by Exponential Linear Units (ELUs)*. arXiv preprint arXiv:1511.07289 (2015).

